# Mood Fluctuation and Psychobiological Instability: The Same Core Functions Are Disrupted by Novel Psychoactive Substances and Established Recreational Drugs

**DOI:** 10.3390/brainsci8030043

**Published:** 2018-03-13

**Authors:** Andrew C. Parrott

**Affiliations:** 1 Department of Psychology, Swansea University, Swansea SA2 8PP, South Wales, UK; a.c.parrott@swansea.ac.uk; Tel.: +44-(0)-1792-295271; 2 Centre for Human Psychopharmacology, Swinburne University, Melbourne, VIC 3122, Australia

**Keywords:** amphetamine, cocaine, mephedrone, cannabis, spice, drug, mood, homeostasis

## Abstract

Many novel psychoactive substances (NPS) have entered the recreational drug scene in recent years, yet the problems they cause are similar to those found with established drugs. This article will debate the psychobiological effects of these newer and more traditional substances. It will show how they disrupt the same core psychobiological functions, so damaging well-being in similar ways. Every psychoactive drug causes mood states to fluctuate. Users feel better on-drug, then feel worse off-drug. The strength of these mood fluctuations is closely related to their addiction potential. Cyclical changes can occur with many other core psychobiological functions, such as information processing and psychomotor speed. Hence the list of drug-related impairments can include: homeostatic imbalance, HPA axis disruption, increased stress, altered sleep patterns, neurohormonal changes, modified brain rhythms, neurocognitive impairments, and greater psychiatric vulnerability. Similar patterns of deficit are found with older drugs such as cocaine, nicotine and cannabis, and newer substances such as 3,4-methylenedioxymethamphetamine (MDMA), mephedrone and spice. All psychoactive drugs damage human well-being through similar basic neuropsychobiological mechanisms.

## 1. Mood State Fluctuations

Psychoactive drugs, by definition, cause mood states to change and fluctuate. Hence an important factor for drug-induced distress is mood instability. This is found with sedative drugs such as alcohol or cannabis, and stimulant drugs such as cocaine or methamphetamine [1,2,3,4,5,6]. Indeed, the main reason that psychoactive drugs are used recreationally is for their “positive” mood effects, such as feelings of relaxation and pleasure [4]. Novel psychoactive substances (NPS) such as mephedrone and spice cannabinoids are very similar in this regard [6,7,8,9]. Although one of the paradoxes of drug taking is that many of the apparent mood gains represent the reversal of unpleasant abstinence feelings. Nicotine is a prime example of this pattern [10,11,12], although it also occurs with other substances [4].

Psychoactive drugs can also produce undesirable mood state changes. For instance, recreational cannabis, whether herbal or spice, can lead to feelings of tension and suspiciousness [2,13]. Stimulants such as cocaine, methamphetamine and mephedrone can also lead to feelings of anxiety and paranoia [1,14,15]. The acute mood effects of all psychoactive drugs can be highly variable and may differ considerably between individuals. On alcohol some users become happy and jovial, while others become moody and aggressive, especially after excess levels of consumption [5,16,17]. In a similar way, Le Strat et al. [18] documented a wide range of responses to an acute dose of cannabis. Furthermore, those with positive initial mood reactions were most likely to become regular cannabis users, whereas those with negative or neutral mood reactions to cannabis tended not to persevere with its usage. Similar differences in mood response have also been empirically demonstrated with the methamphetamine derivative MDMA (3,4-methylenedioxymethamphetamine or “Ecstasy”). This drug is often described as the most euphoriant of all the recreational stimulants. Yet acute MDMA can release a wide range of feelings and cognitions-both positive and negative. Liechti et al. [19] found that an acute dose of MDMA in the laboratory led to significant increases in feelings of both introversion and extraversion, while feelings of happiness and depression were also significantly intensified. In a comprehensive review of the acute-dose MDMA mood literature, it was found that a wide range of positive and negative psychological material could be released/intensified [20]. For instance, psychotherapists who have incorporated MDMA into their therapy sessions have noted that the emergent psychological material can be difficult and stressful for the client to handle, although its release may also be important for potential therapeutic gains [20,21,22,23,24]. In positive environmental situations, the positive mood effects of MDMA predominate, whereas in neutral situations its mood effects can be more variable and less positive [14,15]. These adverse mood abreactions to MDMA can be more frequent than is commonly portrayed. In an article entitled: “Hug drug or thug drug”, Reid et al. [25] noted that MDMA often generated feelings of anger and aggression. Rugani et al. [26] noted that acute psychotic patients who had used MDMA for recreational purposes demonstrated heightened levels of hostility, physical violence, and verbal aggression than non-users. The authors noted that this finding “surely runs counter to the expected entactogenic effects of Ecstasy” (for further debate, see [27]).

In a large Internet study of mephedrone and MDMA users, the acute mood effects of each drug were broadly similar, although some intriguing differences in pharmacodynamic tolerance were apparent [9]. A mixture of positive and negative mood changes is also found with established recreational stimulants such as cocaine [28], and laboratory doses of methamphetamine [14,15]. The effects of high doses of amphetamine and cocaine can be very strong, with reports of a physical rush or hit. These high doses can lead to intensely negative moods, with very severe feelings of suspiciousness, or clinical paranoia. Spice cannabinoids can also have far stronger effects that herbal cannabis, in both positive and negative ways [29]. The more extreme mood reactions of the more powerful drugs can lead to changeable and unpredictable patterns of behavior. However, these abreactions can occur with any psychoactive drug, irrespective of whether they are established or novel.

### 1.1. Drug Withdrawal and Repetitive Mood Vacillation

One of the main problems with every psychoactive drug is that the brief period of on-drug mood gains is followed by a period of neurochemical depletion afterwards, when the opposite mood states develop. These rebound moods are typically negative and aversive, and are readily reversed by taking the drug again. Hence every mood-altering drug has addiction potential. Indeed, the essence of addiction is these repetitive mood vacillations [4,30]. This pattern can be illustrated by legal stimulants such as nicotine, illicit drugs such as cocaine, or novel substances such as MDMA or mephedrone. The former two drugs have been extensively studied, with nicotine showing many pharmacokinetic and pharmacodynamic similarities to cocaine [31]. Both drugs are powerful Central Nervous System (CNS) stimulants, with the first cigarette of the day increasing heart rate substantially [32,33], which is one of the reasons tobacco smokers develop hypertension. In mood terms, tobacco smokers feel more alert after the first cigarette of the day, but soon this activation is lost, and after 20–60 min the regular smoker needs yet another cigarette. This mood fluctuation repeats over the rest of the day and recurs every day for the rest of their lives—until they quit or prematurely die [10,11,34].

Similar patterns of mood fluctuation occur with cocaine, since nasal snorting leads to a rapid hit, followed by a low-mood comedown, and the urgent need for another drug hit. Cocaine therefore displays high addiction potential, with crack cocaine being even more troublesome and addictive, due its extreme rapidity of action [4,35,36]. Khat leaf chewing occurs in many countries around the Horn of Africa [37,38], with cathinone being slowly released into the systemic circulation. Cathinone displays weaker stimulant properties than cocaine. Yet Khat chewers also report acute mood gains, followed by negative moods on withdrawal, in a pattern identical to that found with other stimulants [37]. This is also evident with MDMA, although over a longer time period. Hence the acute moods peak after a couple of hours, while the recovery period can last for several days [28,39,40,41,42]. This long pharmacodynamic profile explains why Ecstasy/MDMA is only taken intermittently and displays lower addiction potential than most other stimulants [43,44].

Cannabis may be a sedative drug, but it shows a similar pattern of mood vacillation to the stimulant drug nicotine. Vandrey et al. [45] compared the mood changes found during withdrawal from cannabis and tobacco, and concluded that they were very similar. For instance, the unpleasant mood effects of cannabis withdrawal could include feelings of irritability, anger, and depression (i.e., very similar to tobacco), along with other problems such as impaired sleep, altered circadian rhythms, changed appetite, and drug cravings [45,46]. These symptoms of cannabis withdrawal have been formalized in standardized questionnaires such as the Cannabis Withdrawal Discomfort Scale [47], and the Marijuana Craving Questionnaire [48]. The high addictiveness of cannabis does make it difficult to understand current movements to make its usage legal [49].

### 1.2. Related Psychobiological Problems

Many other psychological skills and abilities may also fluctuate during drug stimulation and drug withdrawal. For instance, tobacco smokers display worse memories than non-smokers [50]. The reason for this is that new information is being laid down in memory and is being stored under a constantly changing background of nicotine levels. Hence memory storage and retrieval are both adversely affected by nicotine addiction [12]. Sleep is also adversely affected by nicotine addiction, while it improves to normal following smoking cessation [12]. The many psychobiological problems found in recreational stimulant users have been described in numerous comprehensive reviews [1,3,36,51,52].

Similarly, the addictiveness of herbal cannabis was noted earlier, while the stronger skunk varieties of herbal cannabis display greater potential [53]. Artificial spice cannabinoids are even stronger, and hence more damaging. Some of the artificial spices are full agonists for the cannabinoid receptor, whereas herbal cannabis is a weak partial agonist [54]. Hence spice displays far greater addictiveness, with some users committing suicide when they cannot access their normal drug supplies [6,29,55,56,57,58]. The practical consequences of cannabis dependency can be severe [49]. In the USA around 300,000 individuals approach professional drug addiction services for cannabis dependency every year [59]. Clinically disabling dependency occurs in around 10% of those who have ever tried the drug [60], while 65% of cannabis ever-users report some aspects of drug dependency [61], with young initiates the most vulnerable [62]. In summary, the core problems related to drug dependency are similar for stimulants and depressant drugs, and for older and newer psychoactive substances.

## 2. Homeostasis

One fundamental index of psychological balance and health is homeostasis. When the organism is well adapted to its environment, its daily rhythms of behavioral and physiological activity occur smoothly and efficiently. Selye [63] noted that disruption to homeostasis led to psychological imbalance, increased bio-physiological stress, and led to excessive energy expenditure. Furthermore, the repeated experience of acute stress led to cumulative chronic stress and this caused physical and psychological ill-health. Selye [63] showed that the Hypothalamic-Pituitary-Adrenal (HPA) axis was crucial for psychophysiological stability, with cortisol being the key neurohormone for maintaining homeostatic balance [64]. Healthy individuals showed regular circadian rhythms of cortisol secretion, and this master hormone helped to maintain the optimal secretion patterns for other important neurohormones [63,65,66].

Many psychoactive drugs affect neurohormonal secretions acutely, and when these drugs are taken repeatedly, they can lead to chronic stress. This may be illustrated with the recreational stimulant MDMA [65,66]. In placebo-controlled laboratory trials, an acute dose of MDMA can generate a cortisol increase of around 150% [67]. Recreational Ecstasy/MDMA users show peak cortisol increases of around 800%, probably due to the combined effects of taking a stimulant drug in a stimulating environment [42,68]. The Cortisol Awakening Response can also be affected in recreational Ecstasy/MDMA users [69], with around 70% of recreational users complaining of disrupted sleep, even when drug-free [70]. Body temperatures can also change, with MDMA showing well-documented patterns of thermal change [71,72,73]. Synthetic cannabinoids such as AKB48 can induce hypothermia [74], while synthetic cathinones such as mephedrone can also affect thermal reactivity [75]. Returning to chronic stress, regular MDMA users show strong longer-term neurohormonal changes. When cortisol was measured in 3-month hair samples, the regular Ecstasy/MDMA users displayed 400% higher cortisol levels than non-user controls, while the light Ecstasy/MDMA users showed intermediate cortisol values [76]. MDMA is not the only recreational drug which can affect cortisol. Raganathan et al. [77] showed that acute tetrahydrocannabinol (THC) led to a significant increase in cortisol secretion. King et al. [78] found that chronic cannabis users had significantly higher salivary cortisol levels than non-user controls. Currently there is a paucity of empirical evidence on the neurohormonal effects of Novel Psychoactive Substances, and empirical studies in this area are therefore needed.

### Psychiatric Aspects

All psychiatric disorders are dimensional, with symptoms ranging on a continuum from low to high. This core notion may seem rather obvious, but it needs to be stated since it can help explain how psychoactive drugs contribute to mental distress. The core processes described earlier, of mood state vacillation and homeostatic imbalance, can each contribute to mental instability, while those individuals with a predisposition to mental distress may be particularly vulnerable to the destabilizing effects of psychoactive drugs. For an example of this interactive psychiatric model applied to recreational MDMA, see Parrott [79].

The first written report of psychiatric problems being caused by any psychoactive drug were present in the world’s oldest pharmacopoeia, attributed to Emperor Shen Nung in bronze-age China. This noted that when cannabis was taken in excess, it could produce “visions of devils” [80]. Modern research has confirmed that cannabis may cause a form of psychosis, with many similarities to classic schizophrenia [13]. Hence an acute dose of cannabis can induce bizarre thoughts and cognitions [81]. D’Souza and colleagues [82] administered THC and placebo to recreational cannabis users without any prior psychiatric history. The active cannabis condition led to significant increases in scores on the Positive and Negative Symptom Scale (PANSS), the standard rating scale for clinical symptoms of schizophrenia. Individual subjective experiences under the acute influence of THC included the following: “I thought I could see into the future” … “I thought you were trying to program me” … “I thought you could read my mind” … “I thought I was god”. These delusional thoughts as measured by raised PANSS positive symptom scores, can also correlate with changed patterns of brain activity [83].

When used regularly, cannabis can lead to both clinical psychosis, and other forms of psychiatric disorder [84]. The Swedish Conscript study was the first prospective investigation to demonstrate an association between cannabis and schizophrenia [85]. This finding has been replicated in further prospective studies, where regular cannabis use led to an increased risk of psychotic breakdown in later years. Le Bec et al. [86] undertook a comprehensive review and concluded that every published study showed a significant link between recreational cannabis and the later emergence of psychosis. One crucial factor is that the drug needs to be taken repeatedly and regularly. In one prospective study, Henquet et al. [87] found that occasional cannabis users showed no increase in risk, weekly-users showed a slightly increased risk, while daily-cannabis users showed a highly significant increase in later psychosis. Regular cannabis use is also associated with an increased risk for other mental health problems, such as depression and mania [88,89].

Recreational CNS stimulants can also cause greater psychiatric distress. Feyissa and Kelly [90] noted that Khat chewing could lead to a range of mental health problems including depression and hypomania; hence even weaker drugs such as cathinone can lead to psychiatric problems, while regular users of stronger stimulants such as amphetamine, cocaine or methamphetamine can experience a wide range of problems, including psychosis, depression, paranoia, psychomotor tics/tremors, eating disorders, and aggression [1,3,4,35,51,91]. Recreational MDMA is also associated with a wide range of adverse psychiatric consequences, such as clinical depression, aggression, problems with weight control, eating/food intake, and some of these issues may endure for years after drug cessation [26,76,92,93,94,95,96,97,98,99].

## 3. Neurocognitive Deficits

Cognitive skills are an important focus for most areas of applied psychology, and psychopharmacology is no exception. The extensive empirical literature demonstrates both acute and chronic drug influences. By definition, any drug which is psychoactive will affect not only mood states (see Section 1), but many other psychological functions including neurocognition. CNS stimulants such as cocaine or mephedrone will speed information processing, but also increase errors through increased carelessness and impulsivity. CNS depressants will generally lengthen reaction times but may increase errors through reduced alertness and vigilance/attention. When combined with drug-induced feelings of confidence, these changes can make any psychoactive drug dangerous for practical skills such as car driving [4,55,100]. Their chronic use can also be damaging. In an extensive review, Cruickshank and Dyer [1] noted that methamphetamine led to impairments in executive functioning, learning, memory, and motor skills. Other reviews have generated similar lists of neurocognitive impairments following other stimulants such as cocaine [52,101], or Ecstasy/MDMA [27,44,102,103,104,105,106,107,108]. Cannabis can lead to acute deficits in memory, learning, sustained attention, and higher cognitive skills, while its chronic use can lead to a wide range of cognitive deficits, even including a decline in general intelligence, with reduced IQ test scores [109,110,111,112,113,114]. Neuroimaging studies of regular cannabis users indicate deficits in various brain regions, such as the hippocampus and amygdala [114], with white matter degeneration and de-myelination [115].

## 4. Final Overview

Psychoactive drugs can damage human well-being simply by being psychoactive! In acute terms they may boost activity for a short period, but this is soon followed by a period of neurochemical recovery, when the opposite psychological states develop. These psychological fluctuations are readily seen in mood state changes of daily tobacco smokers (see Figure 1 in Parrott, [10]). However, moods and feelings provide just one index for other more general changes in psychological status. Many different psychological functions can be affected—in different ways by different drugs. They also affect many different neurotransmitter systems. Yet despite the multitude and variety of their neurotransmitter actions, in psychobiological terms these drugs all display the same underlying pattern of disrupted balance and equilibrium [4,11,30,38]. These core biological factors also explain why every psychoactive drug displays addiction potential. Regular users suffer from negative states off-drug, and feel better when on-drug, hence the “need” to take the drug repeatedly [10]. As novice users, the more they succumb to their new habit, the stronger their drug dependency becomes. 

Psychoactive drugs also affect the HPA axis, causing hormonal dysregulation, and increasing the susceptibility for stress [4,66]. The healthy human organism displays a natural balance between sympathetic and parasympathetic nervous system activity. So, when humans take recreational drugs, they disturb this natural balance, and this leads to adverse consequences [4]. Proponents for drug use typically only talk about the short-term drug gains, and with this narrow focus, any psychoactive drug can be miss-described as beneficial. It is only by covering all aspects of their acute and chronic usage that a more complete picture of their damaging effects can emerge. 
